# Colorectal liver metastases: surgery versus thermal ablation (COLLISION) – a phase III single-blind prospective randomized controlled trial

**DOI:** 10.1186/s12885-018-4716-8

**Published:** 2018-08-15

**Authors:** Robbert S. Puijk, Alette H. Ruarus, Laurien G. P. H. Vroomen, Aukje A. J. M. van Tilborg, Hester J. Scheffer, Karin Nielsen, Marcus C. de Jong, Jan J. J. de Vries, Babs M. Zonderhuis, Hasan H. Eker, Geert Kazemier, Henk Verheul, Bram B. van der Meijs, Laura van Dam, Natasha Sorgedrager, Veerle M. H. Coupé, Petrousjka M. P. van den Tol, Martijn R. Meijerink, COLLISION Trial Group, Warner Prevoo, Niels Kok, Arjen L. Diederik, Gert Jan Spaargaren, Colin Sietses, Tjarda N. van Heek, Gian Piero Serafino, Jurgen J. Futterer, Peter B. van den Boezem, Martijn Stommel, Hans de Wilt, Mark Arntz, Sjoerd Jenniskens, Mark Besselink, Otto M. van Delden, Thomas M. van Gulik, Pieter J. Tanis, Krijn P. van Lienden, Mark C. Burgmans, Rutger-Jan Swijnenburg, Arian R. van Erkel, Henk H. Hartgrink, Jan Peringa, Hendrik Marsman, Peter C. A. Jacobs, Michael F. Gerhards, Christiaan van der Leij, Rutger Brans, Marielle M. E. Coolsen, Kees C. H. C. Dejong, Ronald van Dam, Abbas Millad Solouki, Johan A. Dol, Ted W. F. Vink, Eric R. Manusama, Gijs A. Patijn, Vincent B. Nieuwenhuijs, Mark A. J. Meijer, Hans Torrenga, Eric D. J. A. Sonneveld, Jan-Willem W. D. de Waard, Joris J. A. Joosten, Cees Verhoef, Adriaan Moelker, Dirk Jan Grunhagen, Bas Groot Koerkamp, Jeroen Hagendoorn, I.Q Molenaar, Rutger C. G. Bruijnen, Karin C. M. J. van Nieuwkerk, Peter van de Ven, Astrid de Wind, Han Anema, Jacob de Bakker, Martijn W. H. Leenders, Tessa Hellingman, Nicole van Grieken, Sanne Nieuwenhuizen, Bart Geboers, David J. Breen, Luca Aldrighetti, Francesco De Cobelli, Francesca Ratti, Paolo Marra, Thomas Albrecht, P. D. Muller, Cornelis van Kuijk

**Affiliations:** 1 Department of Radiology and Nuclear Medicine, Amsterdam University Medical Centres (location VUmc), de Boelelaan 1117, 1081 HV Amsterdam, The Netherlands; 2 Department of Surgery, Amsterdam University Medical Centres (location VUmc), de Boelelaan 1117, 1081 HV Amsterdam, The Netherlands; 3 Department of Medical Oncology, Amsterdam University Medical Centres (location VUmc), de Boelelaan 1117, 1081 HV Amsterdam, The Netherlands; 4 Department of Epidemiology and Biostatistics, Amsterdam University Medical Centres (location VUmc), de Boelelaan 1117, 1081 HV Amsterdam, The Netherlands

**Keywords:** Colorectal cancer, Colorectal liver metastases (CRLM), Liver metastases, Hepatic resection, Liver surgery, Thermal ablation, Radiofrequency ablation (RFA), Microwave ablation (MWA)

## Abstract

**Background:**

Radiofrequency ablation (RFA) and microwave ablation (MWA) are widely accepted techniques to eliminate small unresectable colorectal liver metastases (CRLM). Although previous studies labelled thermal ablation inferior to surgical resection, the apparent selection bias when comparing patients with unresectable disease to surgical candidates, the superior safety profile, and the competitive overall survival results for the more recent reports mandate the setup of a randomized controlled trial. The objective of the COLLISION trial is to prove non-inferiority of thermal ablation compared to hepatic resection in patients with at least one resectable and ablatable CRLM and no extrahepatic disease.

**Methods:**

In this two-arm, single-blind multi-center phase-III clinical trial, six hundred and eighteen patients with at least one CRLM (≤3 cm) will be included to undergo either surgical resection or thermal ablation of appointed target lesion(s) (≤3 cm). Primary endpoint is OS (overall survival, intention-to-treat analysis). Main secondary endpoints are overall disease-free survival (DFS), time to progression (TTP), time to local progression (TTLP), primary and assisted technique efficacy (PTE, ATE), procedural morbidity and mortality, length of hospital stay, assessment of pain and quality of life (QoL), cost-effectiveness ratio (ICER) and quality-adjusted life years (QALY).

**Discussion:**

If thermal ablation proves to be non-inferior in treating lesions ≤3 cm, a switch in treatment-method may lead to a reduction of the post-procedural morbidity and mortality, length of hospital stay and incremental costs without compromising oncological outcome for patients with CRLM.

**Trial registration:**

NCT03088150, January 11th 2017.

## Background

Colorectal cancer is the third most common malignancy worldwide and the second most common cause of cancer related death in developed countries [1, 2]. Approximately half of the patients will develop colorectal liver metastases (CRLM). Only 10–15% are considered eligible for partial hepatectomy (PH), due to (1) an impaired general health status, (2) a history of extensive abdominal surgery, (3) the presence of lesions with an unfavourable anatomical location or (4) an insufficient future liver remnant to resect all lesions [3–7]. These patients are usually treated with chemotherapy and/or thermal ablation, alone or in combination with PH.

Contradictory to most cancer types, long-term survival and even cure is possible in a subset of patients with CRLM [8]. Median overall survival (OS) of untreated CRLM (receiving only symptomatic therapy) is 4.5–12 months [9]. Chemotherapy has improved OS, but OS remains humble at 15–20 months [10, 11].

Surgical resection of the metastases has long been considered the only curative treatment option. In the past few years, radiofrequency ablation (RFA) and microwave ablation (MWA) techniques have rapidly worked their way into clinical guidelines for treatment of unresectable liver tumours [12]. For solitary small (< 2 cm) hepatocellular carcinomas, international guidelines have shifted from surgery to minimally-invasive percutaneous thermal ablation because local control rates have reached 100% [6, 13–17].

Four recent series reported a comparable OS for thermal ablation versus surgical resection [14, 18–20]. These results have led to the discussion whether or not thermal ablation – being less invasive – should be favoured over resection for smaller lesions. Despite this, 5-year OS (25–55%) of thermal ablation for patients with unresectable CRLM has been labelled inferior to surgical resection for patients with resectable CRLM according to previous meta-analyses and systematic reviews [21–29]. These results should be interpreted with caution due to the apparent selection bias. At this point, there are no high-quality randomized controlled trials comparing thermal ablation to surgical resection for resectable CRLM, even though the need has previously been suggested by various authors [8, 30, 31]. To prove non-inferiority, we have designed a two-arm single-blind multi-center phase-III randomized controlled trial comparing surgical resection (standard of care) to thermal ablation (experimental arm) for resectable and ablatable CRLM ≤3 cm.

## Design/methods

### Design

COLLISION is a national, single-blind, multi-center, phase-III trial that is organized by the Amsterdam University Medical Centres (location VUmc) in Amsterdam, the Netherlands. The study is accommodated by the Dutch Colorectal Cancer Group (DCCG) and formally endorsed by the Dutch national covering patient federations, Dutch national societies for interventional radiology (NVIR), radiology (NVvR), surgery (NVvH), and the liver surgery working group (WLC).). Patients will be recruited in, at least sixteen, high-volume centres for liver surgery throughout the Netherlands: Amsterdam UMC (location VUmc), Amsterdam; Amsterdam UMC (location AMC), Amsterdam; Leiden University Medical Center (LUMC), Leiden; Radboud University Medical Center, Nijmegen; Maastricht University Medical Center (MUMC), Maastricht; Antoni van Leeuwenhoek (AvL), Amsterdam; Medical Center Leeuwarden (MCL), Leeuwarden; Ziekenhuis Gelderse Vallei (ZGV), Ede; Isala Klinieken, Zwolle; Deventer Ziekenhuis, Deventer; Westfriesgasthuis, Hoorn; Erasmus Medical Center (EMC), Rotterdam; Jeroen Bosch Ziekenhuis (JBZ), Den Bosch; Medisch Spectrum Twente (MST), Enschede; Onze Lieve Vrouwe Gasthuis (OLVG), Amsterdam; University Medical Center (UMCU), Utrecht). The protocol has been approved by the Medical Ethical Review Board (METc) of the Amsterdam University Medical Centres (location VUmc) for Dutch national approval (no. 2016.561). The trial is investigator-sponsored by Medtronic PLC, independent of industry and registered at clinicaltrials.gov (*NCT03088150*, January 11th 2017). The trial will be conducted in accordance with the Declaration of Helsinki (64th version, October 2013) and the guidelines for Good Clinical Practice (GCP). The in- and exclusion criteria are summarized in Table 1.

**Table 1:** In- and exclusion criteria

Inclusion criteria	Exclusion criteria
Histological documentation of primary colorectal tumour	No target lesions suitable for both resection and ablation
Age > 18 years	Radical treatment unfeasible or unsafe (e.g. insufficient future liver remnant [FLR])
At least one CRLM size ≤3 cm eligible for both surgical resection and thermal ablation (target lesions)	Any surgical resection or focal ablative liver therapy for CRLM prior to inclusion
Additional unresectable CRLM should be ≤3 cm and ablatable	The presence of extrahepatic nodal or non-nodal metastases
Additional unablatable CRLM should be resectable	Immunotherapy ≤6 weeks prior to the procedure
Maximum number of CRLM 10	Chemotherapy ≤6 weeks prior to the procedure
Resection for resectable lesions considered possible obtaining negative resection margins (R0) and preserving adequate liver reserve	Pregnant or breast-feeding subjects. Women of childbearing potential must have a negative pregnancy test performed within 7 days of the start of treatment
Resectability and ablatability should be re-confirmed by intra-operative ultrasound (IOUS) and full surgical exploration	Compromised liver function (e.g. signs of portal hypertension, INR > 1,5 without use of anticoagulants, ascites)
Eastern Cooperative Oncology Group status (ECOG) 0–2	Uncontrolled infections (> grade 2 NCI-CTC version 3.0)
American Society of Anesthesiologists (ASA) grade 1–3	Severe allergy to contrast media not controlled with premedication
Life expectancy of at least 12 weeks	Any condition that is unstable or that could jeopardize the safety of the subject and their compliance in the study;
Adequate bone marrow, liver, and renal function as assessed by local usual laboratory tests. As usual, these results should be judged by the local investigator and should be conducted within 7 days prior to definite inclusion. Written informed consent	Substance abuse, medical, psychological or social conditions

**Fig. 1:**
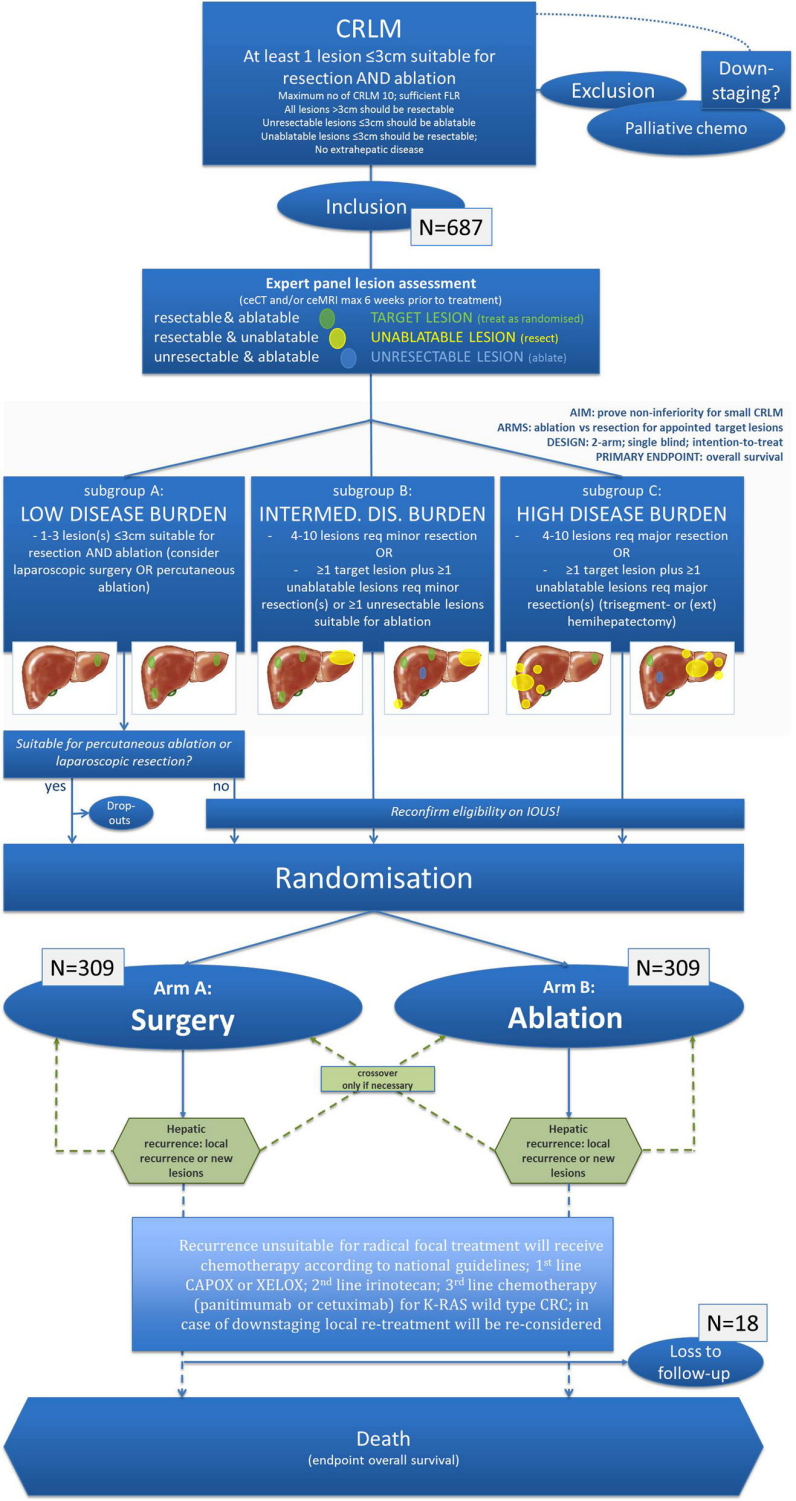
Flow diagram of study procedure

The total duration of the study is around 13 years considering an inclusion time of 3 years and a minimum follow-up period of 10 years. All participants will provide written informed consent.The flow diagram of is shown in Fig. 1.

#### Start COLLISION trial

Inclusion, randomization and treatments started in two hospitals by the end of 2017. Due to formal approval procedures by local authorities, only AmsterdamUMC (location VUmc) and ZGV Ede were able to include patients from the beginning. From May 2018, RadboudUMC, LUMC, MCL, Isala Klinieken and Westfries Gasthuis were also able to participate. Numerous other Dutch high-volume liver centres, which are mentioned above, are waiting for local approval and will participate in the near future.

#### Eligibility

Potential candidates will be registered and undergo routine pre-procedural work-up: baseline full blood examination, carcinoembryonic antigen (CEA), bone marrow, liver, and renal function -, anaesthetic review, ceCT of the chest and abdomen and either an upper abdominal ceMRI or a total body ^18^F-FDG PET-CT using upper abdominal ceMRI as problem solver. Patients with ≥1 resectable and ablatable CRLM (≤3 cm), no extrahepatic disease and a good performance status (WHO 0–2) are considered eligible. Supplementary resections for resectable lesions > 3 cm and thermal ablations for unresectable CRLM ≤3 cm are allowed with a maximum number of CRLM of 10 (Table 1).

Eligible patients will be stratified into low-, intermediate- and high disease burden after assessment by an expert panel (Fig. 1). The panel, consisting of at least two diagnostic radiologists, two interventional radiologists and two hepatobiliary and/or oncological surgeons, will appoint lesions that are resectable and ablatable as target lesions, resectable and unablatable lesions as unablatable lesions and ablatable but unresectable lesions as unresectable lesions. All unablatable lesions should be resectable and all unresectable lesions should be ≤3 cm and ablatable. Because definitions of resectability and ablatability can vary dramatically from one center to the other and from one specialist to the other, the panel has to agree with the treating physicians’ treatment plan. If the panel disagrees, the panel and the treating physicians must reach consensus before the patient can be enrolled.

### Methods

Participating centres should have extensive experience in the field of both hepatic surgery and thermal liver tumour ablation, defined as performing ≥20 procedures annually. Treating surgeons and interventional radiologists should be board certified and have performed and/or supervised ≥100 procedures.

#### Inclusion

After having obtained written informed consent by the outpatient clinic doctor, patients will be formally included. The patient should be scheduled to undergo the procedure within a time-frame of maximum 6 weeks hereafter. Patients suitable for either laparoscopic resection or percutaneous ablation (Subgroup A, low disease burden; 1–3 target lesions) will be randomized prior to the procedure. All other patients will undergo open laparotomy with surgical inspection of the abdominal cavity and intra-operative ultrasound (IOUS).

#### Exclusion (drop-outs)

Despite improvements in preoperative imaging technology, the intraoperative use of ultrasonography remains of crucial importance [32]. The detection rate of preoperatively unknown lesions is still high (up to 50%) with considerable consequences on treatment strategy [32]. Following surgical inspection and IOUS the inclusion criteria need to be reconfirmed prior to randomization. If (1) a radical procedure is no longer considered safe or feasible, if (2) > 10 CRLM are present, if (3) extrahepatic disease is detected, or if (4) no lesion can be appointed as target lesion, the patient cannot be included in the study and will be treated as non-study object. Additional CRLM suitable for both resection and ablation ≤3 cm will be appointed as new target lesions. Additional unresectable lesions ≤3 cm that are suitable for thermal ablation should be ablated if possible and vice versa additionally detected unablatable lesions should be resected. Lesions, preprocedurally appointed as target lesions, that prove unsuitable for one treatment modality based on IOUS lose their status and should be treated with the alternate modality (lesion shifts from target lesion to unablatable or unresectable lesion).

#### Laparotomy

The surgical explorative procedure of participants in this study is identical to standard procedures for non-study objects. A right subcostal incision is performed. The abdominal cavity will be explored in order to exclude extrahepatic tumour manifestations. An IOUS to exclude additional CRLM and for final confirmation of resectability will always be performed.

#### Randomization

Patients with limited disease burden (max. 3 lesions ≤3 cm) that are suitable for percutaneous ablation or laparoscopic resection will be randomized, prior to the procedure, into one of two arms, arm A and arm B. All other patients will undergo laparotomy with IOUS and surgical inspection and will, if still considered eligible, be randomized during general anaesthesia. Patients included in study arm A will undergo resection of hepatic metastases, allowing thermal ablation for additional unresectable lesions. Patients included in study arm B will undergo ultrasound guided thermal ablation of hepatic metastases, allowing resection for additional unablatable lesions (Fig. 1).

Randomization is centralized and performed through a web-based module (Castor EDC®) [33], which is accessible 7 days a week, 24 h per day]. For open procedures randomization will be performed shortly after surgical inspection and IOUS with the patient under general anaesthesia. Both the experimenter(s) and the participant will be unaware of the eventual treatment arm prior to the procedure; after the procedure the patient will remain unaware (single-blind).Because follow-up imaging will reveal the nature of the focal therapy and because knowledge about the actual procedure and pathological confirmation of tumour free margins is required to reliably assess ^18^F-FDG PET-CT follow-up scans, the panel’s diagnostic abdominal radiologists and nuclear physicians need to be informed about the specific treatment history.

Changes in insights detected after randomization do not allow patient’s exclusion. These patients will remain in their originally appointed group according to the intention-to-treat analysis. For example, if, after being randomized into the resection arm, a target lesion proves unresectable during surgical tissue preparation and dissection, the patient will remain in arm A (resection) even if the lesion was eventually ablated or left untreated.

#### Surgical resection

In case of randomization to surgical resection, the surgeon will remove all target lesions as well as all additional unablatable lesions. The extent of the resection, the resection margins and the specific technique is at the discretion of the performing liver surgeon. Complications encountered during the procedure will be noted. Postoperative care will be on the recovery and subsequently on either the surgery ward or medium care whenever deemed necessary. General ‘resectability’ criteria are shown in Table 2.

**Table 2:** General ‘resectability’ and ‘ablatability’ criteria

General ‘resectability criteria’	General ‘ablatability criteria’
No size limit	Maximum CRLM size ≤3 cm
Aiming at negative (R0) margins	Aiming at a tumour free margin of > 10 mm
Leave sufficient FLR (> 20% normal functioning liver parenchyma; > 30% post-chemotherapy)	Leave sufficient FLR (> 20% normal functioning liver parenchyma; > 30% post-chemotherapy)
Portal vein embolization of the (most) affected liver lobe may be considered for patients with insufficient FLR	To preserve the major bile ducts (common, right and left hepatic duct) a minimum distance (lesion to major bile duct) of 15 mm is required
At least one of three hepatic veins should be preserved and both the portal venous and hepatic arterial blood flow in the future liver remnant should be remain unharmed	Radical ablation(s) with or without surgical resections for additional unablatable lesions
Approachable surgical field, without extensive scar formation, major surgical adhesions and/or intestinal herniations (risk of major morbidity estimated > 20%; risk of mortality estimated > 5%)	To avoid collateral damage to the intestines a minimum distance to the stomach, small bowel and colon of 15 mm should be pursued in open procedures and respected in percutaneous procedures; Pneumo- or hydrodissections to shift bowels are allowed
Maximum total number of CRLM 10	Maximum total number of CRLM 10

#### Thermal ablation

The safety, feasibility and preferred type of thermal ablation(s) is at the discretion of the interventional radiologist. Ablations are performed according to the CIRSE quality improvement guidelines with an intentional tumour free ablation margin of at least 1 cm [34].

Patients with limited disease burden (max. 3 lesions ≤3 cm) and no contra-indications for a percutaneous approach will be randomized prior to the procedure. Contra-indications for a percutaneous approach are proximity of critical structures. To avoid collateral damage to intestines a minimum distance to the stomach, small bowel and colon of 15 mm should be respected. Laparoscopic approach is allowed. Pneumo- and hydrodissections are allowed. Pringle-manoeuvres are not allowed.

Following percutaneous ablations, a ceCT or ceMRI should always be performed for ablated lesions > 2 cm and for lesions 0–2 cm with radiologically unclear margins after the ablation. Unequivocal local site residues or insufficient tumour-free margins should be re-ablated (completion ablation) within 4 weeks after the initial ablation. If re-ablated within 4 weeks, the residue/insufficient margins count as technically unsuccessful ablations, but not as a tumour recurring event when assessing the primary technique efficacy, local progression-free and disease-free survival. Patients with limited disease burden plus a contra-indication for both percutaneous ablation and for laparoscopic surgery and patients with intermediate or high disease burden will be randomized during open laparotomy.

The probes are connected to compatible and commercially available generators. Ablations will be performed according to the protocols provided by the manufacturers. If necessary, the needle electrodes will be repositioned for one or more overlapping ablations. The proximity of a large portal or systemic vein or hepatic artery is no contraindication for performing the thermal ablation.

The definition of a technically successful ablation is based upon the specific protocols established by the device manufacturers in combination with an immediate post-procedurally performed US (fully hyperechoic ablation zone with an intentional margin of at least 1 cm) [7]. Necessity for re-ablations and/or needle repositioning will be judged by the performing interventional radiologist. Postoperative care will be on the recovery room and subsequently on either the surgery ward or medium care whenever deemed necessary. A quality-control ceCT can be performed within 1–6 weeks after the initial treatment to assess for a completion-procedure [7]. General ‘ablatability’ criteria are shown in Table 2.

#### Follow-up

Conferring to national guidelines follow-up will include imaging, laboratory tests including tumour markers (CEA) and clinical examination every 3 months for the first year and every 6 months hereafter. Follow-up cross-sectional imaging should include at least an abdominal ceCT or upper abdominal ceMRI at the given time-points. Participating centres are free to add ^18^F-FDG PET-CTs at specific time-points or to use alternating specific modalities, as long as the follow-up protocol is pre-approved by the trial coordinators and as long as follow-up imaging is identical for both treatment arms. Quality of life questionnaires will be assessed at baseline, every 3 months for the first year and every 6 months hereafter accordingly. Data will be collected in Castor EDC® [33], only available for related research investigators.

#### Primary and secondary objectives

The main objective is to prove non-inferiority of thermal ablation compared to hepatic resection in patients with at least one resectable and ablatable CRLM (≤3 cm) and no extrahepatic disease. Primary endpoint is OS. Main secondary endpoints are overall disease-free survival (DFS), time-to-progression (TTP), time-to-local-progression (TTLP), primary and assisted technique efficacy (PTE, ATE), procedural morbidity and mortality, length of hospital stay, assessment of pain and quality of life (QoL), cost-effectiveness ratio (ICER) and quality-adjusted life years (QALY).

Pain analysis will be performed using visual analogue scale questionnaires (VAS) assessed prior to, directly after and every 3 months after local treatment; administered pain medication will be registered. Quality of life analysis will be performed using European Organisation for Research and Treatment of Cancer Quality of Life questionnaires (EORTC-QLQ-CR29, EORCT QLQ-C30, EQ-5D) prior to, and every 3 months after local treatment. Patients who complete the quality-of life questionnaires at baseline and at least once during treatment and follow-up will be included in the analysis. The largest decrease in quality of life with respect to baseline will be calculated. The Wilcoxon rank sum test will be used to detect statistical differences between the two treatment arms.

#### Sample size calculation and statistical considerations

We hypothesize (null-hypothesis) that thermal ablation is non-inferior to surgical resection for the selected patient groups in terms of the primary objective (OS). The Cox proportional hazards model (1-sided; non-inferiority or superiority) is used for sample size calculations (Table 3). Given the superior safety profile we consider a hazard ratio (HR) of 1.3 to represent the upper limit of non-inferiority (non-inferiority margin). An HR of 1.3 corresponds to a 56.5% chance of the ablated patients to die first ((P = HR/(1 + HR) = 1.3/(1 + 1.3) = 0.565 (56.5%)). We will have reached 60% of events (death) approximately 6.5 years after having included the last patient (overall probability of event, pE = 0.6). The calculated sample size therefore is 599 (NS). To account for a 10% drop-out ratio (NDO = 69) prior to randomization and a 3% loss to follow-up (NLTFU = 18) after randomization we need to recruit 687 patients (NI). A total number of 618 patients (687–69 (NDO)) will be randomized (NR) into one of two arms: arm A will undergo surgical resection (*n* = 309) and arm B thermal ablation (n = 309) for appointed target lesions.

**Table 3:** Sample size calculation

Significance level (α)	0.05
Power (1-β)	0.80
Hazard Ratio (HR), θ (non-inferiority margin)	1.3
Null-Hypothesis Hazard Ratio, θ_0_	1.0
Recruitment time/study accrual (months)	36
Follow-up time (months)	60
Ratio control vs. experimental: m_2_/m_1_	1.0
Total sample size (N_S_)/total number to be randomized (N_R_)	599
Accounting for 3% loss to follow-up after randomization (N_LTFU_)	18
Accounting for 10% drop-out ratio pre-randomization (N_DO_)	69
Initial pre-randomization sample size – number of included patients (N_I_)	687

#### Statistical methods

All clinicopathological and procedural variables will be described and analysed. Continuous variables will be summarized with standard statistics including, means, standard deviations, medians and ranges. Categorical variables will be summarized with frequencies. When appropriate, box plots and cross tables will be used for descriptive statistics of continuous and categorical variables, respectively. *P*-values below 0.05 will be considered significant. All calculations will be generated by statistical package for social sciences software (SPSS®). Calculation of the number of patients that will be needed to address our primary endpoint with a power of 80% and a 5% type I error rate is described in the sample size calculation section.

Univariate survival analysis will be performed using the Kaplan-Meier method. Differences in survival lengths will be analysed using the log rank test. To determine hazard ratios (HR) for multivariate analysis, Cox regression will be used. Significance of differences for continuous and categorical data will be analysed using the Mann-Whitney U test and Chi-square test respectively. When appropriate, box plots and cross tables will be used for descriptive statistics of continuous and categorical variables, respectively. OS will be estimated by the Kaplan-Meier method with corresponding two sided 95% Cl’s for survival proportions.

Primary and assisted technique efficacy rates (PTE, ATE) defined as the percentage of target lesions that have recurred after the initial local treatment and after additional local treatments regardless of the technique(s) used to treat the recurrence with a minimum follow-up period of 12 months after the last focal therapy;- Direct and indirect total cost of care for both treatment arms will be registered in the cost-effectiveness data collection matrix. Based on this matrix a cost–utility analysis, measured in terms of years of full health lived, using quality-adjusted life years will be prospectively calculated. Cost-effectiveness will be expressed as an ICER, the ratio of change in costs to the change in effects.

#### Data monitoring

The investigators believe that an independent data safety and monitoring committee (DSMB) is unnecessary given the much less invasive nature and superior safety profile of the experimental treatment arm (thermal ablation). An independent monitor committee (Clinical Research Bureau; CRB) is appointed to safeguard the quality of all investigator-initiated studies. A quality officer from the CRB will monitor all study data according to Good Clinical Practice (GCP). The informed consent of selected individual participants will be checked. Source Data verification will be performed during onsite monitoring (to verify if all data on the Case Report Form are in accordance with the source data). The intensity of this verification is in relation to the risk associated with the intervention investigated, which is considered acceptable. For all subjects, the informed consent forms, the in- and exclusion criteria and the primary outcome (overall survival from the date of randomization to the date of death due to any cause) will be verified. The monitor will also verify if all (S)AE’s are reported adequately and within the time that is determined by legal rules and regulations.

Shortly after beginning of the study the research group and epidemiologists will compose a detailed plan regarding futility and criteria to end the study prematurely. The interim analysis will be performed on the primary endpoint using a non-inferiority analysis. If at interim analysis, after having randomized 30% of the patients, the number of deaths due to treatment is significantly higher in patients included in the experimental arm B compared to patients included in the control arm A, the study will be ended prematurely. If the interim analysis shows a trend towards a type 1 or type 2 error, we will add a Data Safety and Monitoring Board (DSMB) to our study. A new interim analysis will be conducted after having randomized 50% of the patients.

#### (Serious) adverse events (AE’s and SAE’s) and serious adverse device effects (SADE)

All serious adverse events that occur in the first 90-days after the procedure that are life threatening or result in death, both related and unrelated to the research, and serious adverse events that happen during complete study follow up, that are life threatening or result in death and are related (unlikely, possible, probable or definite) to the research according to one of the principal investigator, will be reported within 7 days after the responsible investigator has first knowledge of the adverse reaction. This is for a preliminary report with another 8 days for completion of the report. Relationship of the event to the research will be established by the primary investigator as: 1 = Unrelated (clearly not related to the research), 2 = Unlikely (doubtfully related to the research), 3 = Possible (may be related to the research), 4 = Probable (likely related to the research), 5 = Definite (clearly related to the research). All participating clinicians will be made aware of the necessity to report (serious) adverse events to the principal investigators. The sponsor will report the SA(D)E’s through the web portal *ToetsingOnline.nl* to the accredited EC that approved the protocol, within 15 days after the sponsor has first knowledge of the serious adverse events.

The expedited reporting will occur not later than 7 days after the responsible investigator has first knowledge of the adverse event. This is for a preliminary report with another 8 days for completion of the report.

The sponsor (also) has an insurance which is in accordance with the legal requirements in the Netherlands (Article 7 WMO and the Measure regarding Compulsory Insurance for Clinical Research in Humans of July 1st, 2015). This insurance provides cover for damage to research subjects through injury or death related to study participation.

#### Cost-effectiveness analysis

##### General considerations

For this clinical trial, a cost effectiveness (utility) analysis will be performed from a societal perspective, using a 3-year time horizon. The direct and indirect costs will be included. Direct costs taken into account will include treatment costs, cost of hospitalization, medication, imaging, laboratory testing and pathology.

Within the trial, resource use will be monitored and this will be linked to integral cost prices or Dutch tariffs.

##### Patient outcome analysis

To assess indirect cost, patients will be asked to fill out the Productivity and Disease Questionnaire (PRODISC) every 6 months. To calculate total indirect costs, the friction cost approach will be used.

##### Cost analysis

the primary health outcome measure in this economic evaluation will be the total quality adjusted life years (QALY) per trial arm. QALYS will be calculated by using the utility scores linked to the various health states of the EQ-5D; in essence the length of time a patient spends in a particular health condition is weighed by the corresponding utility. Missing data on costs and utilities will be imputed using multiple imputation. The difference in total costs and total QALYs in both arms will be used to calculate the incremental cost-effectiveness ratio (ICER): the cost per QALY gained (or cost-savings per QALY gained or lost), using the formula: ICER = (Cintervention - Ccontrol)/(QALYintervention - QALYcontrol). Cost and health effect will be discounted using the Dutch discount rates of 1.5% for health effects and 4% for costs. In addition, to allow comparison with international studies, discount rates of 3% for both health effects and costs will be used as well. To assess the impact of uncertainty, a probabilistic sensitivity analysis will be performed using the non-parametric bootstrap with 5000 replications. The results will be presented on cost-effectiveness planes. In addition, ICER acceptability curves will be presented and univariate sensitivity analyses will be performed focusing on uncertainty around most important costs-items.

#### Dissemination policy

To ensure optimal implementation we used the framework of *Fleuren* et al. [35]; consisting of patient, innovation, organization and socio-political determinants. Although clinical equipoise between surgery and ablation is reached for small CRLM, the results from recent meta-analyses, such as the most recent one by Meijerink et al. [36], do not support thermal ablation for resectable CRLM outside clinical trials. Hence, patients suitable for COLLISION will have to choose between surgery (+/− ablation for unresectable CRLM) and trial participation.

With 15.549 new cases of colorectal cancer in the Netherlands (2015) approximately 4% of them will have ≥1 resectable and ablatable CRLM [1]. At this moment, these lesions are treated by resection, whilst ablation may be associated with less complications and an equal or even superior oncological outcome. In other words, in the Netherlands alone an estimated target population of 625 patients per year should be eligible for COLLISION trial participation. To further facilitate implementation the trial is formally supported by the following concerning patient federations who joined the trial advisory board: The Dutch Federation for patients with cancer (NFK), the Dutch society for patients with gastro-intestinal and hepato-, pancreatico-, biliary cancers (SPKS) and the Dutch society for image-guided treatment of cancer (SBBvK).

The study is embedded within the multidisciplinary Dutch Colorectal Cancer Group (DCCG). DCCG is a collaboration between medical disciplines that are relevant for the diagnosis and treatment of colorectal cancer (surgical oncology, radiotherapy, medical oncology, pathology, radiology, gastroenterology, genetics). Patients will be recruited throughout the country and treated in one of the qualifying and selected high-volume centres. We will ensure that the scientific community, patients and professional organizations will be constantly kept up to date on the obtained results.

In order to qualify for reimbursement the Dutch health care institute (ZiNL) demands the best available evidence. Currently, thermal ablation is only approved for truly unresectable and small CRLM. Outside the setting of the trial ablations of resectable CRLM are off-guideline and hence not reimbursed. The direct and indirect costs of thermal ablation are considerably lower than that of surgery. We expect even lower indirect costs for patients treated within the study, primarily because thermal ablation of resectable CRLM in patients who by definition qualify as suitable for surgery may be associated with an even lower complication-rate.

## Discussion

The recently published primary efficacy rates (complete ablation after the first procedure) of RFA and MWA for small CRLM have approached the reported resection plane recurrence rates for similar sized lesions [6]^,^ [14–17], [21]. Hence the issue of ablation site recurrences, that has previously prevented its widespread adoption, may be outdated. The relative ease to percutaneously re-ablate potential site recurrences, nowadays in the setting of a one-day admission under conscious sedation, has further downgraded its relevance.

### Partial hepatectomy

Until relatively recent, patients with CRLM could only be cured by surgical resection of the lesions. Although no formal upper limit regarding number and size of CRLM has been established, surgical resection is nowadays considered safe and effective for patients with an adequate performance status if radical resection will leave sufficient future functioning liver parenchyma. In addition, one of the three main hepatic veins must be uncompromised and the liver remnant has to comprise a portal vein, hepatic artery and a bile duct. Clear definitions of what is regarded as resectable are lacking and vary dramatically from center to center on the basis of aggressiveness of the surgical team and the perception of the medical oncologist on when to refer patients [37]. To achieve consensus several societies for surgical oncology and hepatobiliary surgery have previously attempted to postulate resectability criteria (Table 2) [38, 39]. The objective of surgical resection for CRLM should be to remove all macroscopically visible tumour tissue with the intent to achieve cure. Histological tumour free margins and hence the confirmation of having radically resected the metastases remains essential.

Surgical resection has a 5-year OS reaching 31–58% [3, 40]. Although the number of serious adverse events of hepatic resection has decreased considerably in the past two decades, the 90-day mortality (4%) and the complication-rate (40%; major plus minor) are still high [41–43]. In 2007 data from 1059 non-cirrhotic patients who underwent major hepatectomy were analysed [43]. The total percentage of complications was 453 (43%), divided as follows: minor complications 26% (grade I 7%; grade II 19%) and major complications 17% (grade IIIa 10%, grade IIIb 2%, grade IVa 4%, grade IVb 1%). Most frequently encountered complications include per-operative major bleeding, bile duct/gallbladder injury, perforation of adjacent structures, intra-abdominal infection, wound infection, liver abscess, haematoma at incision site, pneumothorax, liver failure and death (4%) [41, 42].

### Radiofrequency ablation

Since its introduction in the late 90’s, RFA is the most studied and widely adopted ablative technique. It has emerged as a promising approach in the treatment of patients with unresectable CRLM. RFA has acquired its role in the treatment of patients with unresectable CRLM as a safe, well tolerated, easily repeatable and less invasive procedure [44, 45].

One major drawback of RFA is the heat-sink effect in highly perfused organs, such as the liver where a large tumour located near large vessels (> 3 mm diameter) is not properly treated because heat is lost to the flowing blood. Another risk of RFA is heat injury to vital structures in or surrounding the ablated area. For this reason, treatment of lesions in the proximity of other organs, large vessels and major bile ducts has to be performed with caution, and is sometimes contra-indicated [46].

The 90-day mortality of thermal ablation alone is very low (< 1%) and the complication rate is also low (6–9%) [6]. Applied to unresectable CRLM, 5-year survival rates are approaching the results reported after surgical resection, especially for patients presenting with a limited number of small-size lesions. The reported 5-year OS is 25–55% [21–29]. The recently presented long-term results from the only available randomized controlled trial shows a survival plateau of 36% after 8-years in patients with unresectable CRLM [6]. It is important to realize that these percentages are derived from studies where thermal ablation was used to treat unresectable lesions. Ruers et al. found a PFS of 16.9 months (95%CI 11.7–22.1) in a group of patients who received chemotherapy plus RFA (HR 0.63 [95%CI 0.42–0.95]). Of those 56 patients treated with RFA, 9 developed a local site recurrence (LSR) (16,1%) [6].

Complications can be divided into three different groups: related to probe placement (bleeding 0,7%, infection, tumour seeding 0–0,3%), related to energy delivery (damage to bowel, gallbladder, bile ducts 4,2%, grounding pad burns, post-ablation syndrome, hepatic vascular damage, liver failure 2,1%) and related to the general procedure (deep venous thrombosis, pulmonary embolism, referred pain, fever, nausea, vomiting, kidney failure) [47].

### Microwave ablation

MWA is known as ablative technique for tissues with a high percentage of water and has several theoretical advantages that may result in improved performance near blood vessels. Due to a much broader field of power density, MWA results in a larger zone of active heating. This increased zone allows for a more homogeneous zone of tumour cell death, both within the targeted zone and next to blood vessels. This feature is thought to make MWA less affected by heat sink. Recent developments in the field of MWA, employing higher frequency bands (2.45 GHz) or spatial energy control (thermal, field, and wavelength), claim to create more predictable, larger and more spherical ablation zones regardless of target location, tissue type or changes in tissue properties during the ablation [48].

Several studies reported a 3-,4- and 5-year OS for MWA between 35 and 79%, 35–58% and 17–18% [15, 16, 49–54]. M7ortality is ranging between 0 and 2% [15, 49, 50]. The median DFS ranges between 8 and 12 months [15, 50, 54]. Overall recurrence ranges between 39 and 72% [15, 17, 50, 51, 55, 56]. In several observational studies complications ranged between 0 and 54% [15, 16, 50, 55–57]. No studies reported the effect on quality of life after MWA.

### Partial hepatectomy versus thermal ablation

Numerous studies reported OS rates for surgery and thermal ablation techniques. Comparing RFA alone to surgery alone numerous observational studies reported corrected hazard ratios for OS between RFA and surgery alone; treatment with RFA was associated with an inferior OS (HR = 1.92; 95%CI 1.44–2.56) [22, 23, 25, 27, 58–61]. Comparing RFA plus surgery to surgery alone other studies reported corrected hazard ratios and allowed for pooling between surgery and surgery plus RFA; no significant difference in OS was found (HR = 1.29; 95% CI 0.71–2.327) [14, 18, 61, 62].

For MWA, a 3-year OS of 23% after surgery and 14% after MWA has been reported [54]. Another study showed a 4-year OS of 70% after surgery and 41% after MWA, although no formal statistical comparison with surgery alone was reported [53]. A more recently published study found 5-year OS rates for surgery versus percutaneous ablation as first intervention of 51.9 and 53%, with a median OS of 65.0 (95%CI 47.3 to 82.6) and 62.1 (95%CI 52.2 to 72.1) months, respectively [19].

Another study reported no significant difference in OS for MWA plus surgery versus surgery alone (3-year OS: 50.9% vs 48.8%) [63]. Median OS was 39 months after surgery and 28 months after MWA plus surgery. In multivariate analysis MWA was no prognostic factor for OS.

Several studies revealed that complications were significantly more common after surgery compared to RFA (relative risk [RR] = 0.47; 95%CI 0.28–0.78) [22–29, 59, 64–66]. Two studies reported serious adverse events in 21–28% after surgery vs 13–37% in the surgery + ablation group [18, 24].

Some studies compared RFA to surgery alone regarding local progression-free survival (LPFS) and DFS; RFA was inferior to surgical resection (+/− RFA) [25, 28, 58]. Comparing RFA plus surgery to surgery alone, RFA plus surgery was associated with a poor LPFS [14, 18, 24, 58, 61]. Assessing DFS, no significant difference between RFA + surgery vs surgery alone was found.

In conclusion, a recently published systematic review and meta-analysis reported that further randomized assessments of thermal ablation with curative intent to current-day palliative chemotherapy alone should be considered unethical [36]. Therefore, the highest achievable evidence level for unresectable CRLM seems to be reached. According to above mentioned superior safety profile, lower complication-rate and competitive long term survival after thermal ablation for CRLM challenges liver surgery and fiats the setup of this randomized controlled trial. If thermal ablation for resectable CRLM proves to be non-inferior to surgery, a reduction of the post-procedural morbidity and mortality, length of hospital stay and incremental costs can be expected, with better quality of life and without compromising oncological outcome.

## Abbreviations

^18^F-FDG PET-CT: fludeoxyglucose F 18 positron emission tomography–computed tomography
AE: Adverse events
ALT: Alanine transaminase
ASA: American Society of Anaesthesiologists
AST: Aspartate aminotransferase
ATE: Assisted technique efficacy
CEA: Carcinoembryonic antigen
Ce-CT: Contrast-enhanced computed tomography
Ce-MRI: Contrast-enhanced Magnetic Resonance Imaging
CRB: Clinical research bureau
CRLM: Colorectal liver metastases
CRS: Clinical risk score
DCCG: Dutch Colorectal Cancer Group
DFS: Disease-free survival
DSMB: Data safety monitoring board
ECOG: Eastern Cooperative Oncology Group status
FLR: Future liver remnant
GCP: Good clinical practice
GHz: Gigahertz
HR: Hazard ratio
ICER: Incremental cost-effectiveness ratio
INR: International normalized ratio of prothrombin time of blood coagulation
IOUS: Intra-operative ultrasound
LPFS: Local progression-free survival
LSR: Local site recurrence
MWA: Microwave ablation
NDO: Number of drop-out
NI: Number of included patients
NLTFU: Number lost to follow-up
NR: Total number to be randomized
NS: Total sample size
NVIR: Dutch national society for interventional radiology
NVvR: Dutch national society for radiology
OS: Overall survival
pE: Probability of event
PH: Partial hepatectomy
PTE: Primary technique efficacy
QALY: Quality-adjusted life years
QoL: Quality of life
RFA: Radiofrequency ablation
RR: Relative risk
SADE: Serious adverse device effect
SAE: Serious adverse events
TTLP: Time-to-local-progression
TTP: Time-to-progression
ULN: Upper limit
VAS: Visual analogue scale
WHO: World Health Organization
WLC: liver surgery working group
